# Cilioretinal Arteries in Highly Myopic Eyes: A Photographic Classification System and Its Association With Myopic Macular Degeneration

**DOI:** 10.3389/fmed.2020.595544

**Published:** 2020-12-02

**Authors:** Jiaqi Meng, Ling Wei, Keke Zhang, Wenwen He, Yi Lu, Xiangjia Zhu

**Affiliations:** ^1^Eye Institute and Department of Ophthalmology, Eye and ENT Hospital, Fudan University, Shanghai, China; ^2^National Health Commission Key Laboratory of Myopia (Fudan University), Shanghai, China; ^3^Key Laboratory of Myopia, Chinese Academy of Medical Sciences, Shanghai, China; ^4^Shanghai Key Laboratory of Visual Impairment and Restoration, Shanghai, China

**Keywords:** cilioretinal artery, myopic macular degeneration, high myopia, fundus photography, visual acuity

## Abstract

**Purpose:** To develop a photographic classification for cilioretinal arteries and to investigate its association with myopic macular degeneration (MMD).

**Methods:** One thousand six hundred ninety-two highly myopic eyes of 1,692 patients were included. The presence of a cilioretinal artery was determined by fundus photographs, and a photographic classification was proposed. MMD was classified according to the International META-PM Classification. Associations of the cilioretinal artery and its classifications with MMD and visual acuity were analyzed.

**Results:** Of the eyes tested, 245 (14.5%) had a cilioretinal artery. The cilioretinal arteries were classified into four categories (temporal “cake-fork,” 35.92%; temporal “ribbon,” 53.47%; “multiple,” 6.53%; “nasal,” 4.08%) and 3 distributions based on whether its visible branches reached the central foveal area. Eyes with cilioretinal arteries had significantly less MMD of grade ≥3 and better visual acuity than those without (*P* < 0.01). Multiple linear regression analysis showed that younger age, male sex, shorter axial length, and the presence of a cilioretinal artery were associated with better visual acuity in highly myopic eyes (all *P* < 0.05). The “nasal” category presented more MMD with grade ≥3 and worse visual acuity than the other categories (*P* < 0.05), whereas the “multiple” category contained no eyes with MMD grade ≥3. The cilioretinal arteries reaching the central foveal area showed less MMD of grade ≥3 and better visual acuity than those not (*P* < 0.05).

**Conclusions:** We propose a photographic classification for cilioretinal arteries that has good clinical relevance to visual functions. The cilioretinal artery may potentially afford protection against MMD.

## Introduction

With the increasing prevalence of high myopia (1), myopic macular degeneration (MMD) has become one of the major causes of visual impairment worldwide (2, 3). It is characterized by a range of degenerative lesions in the retina and choroid, secondary to the increase in axial length (AL) and the formation of staphyloma (4). According to the International Meta-Analysis for Pathologic Myopia (META-PM) Classification System (5), the severity of MMD can be classified into four grades: tessellated fundus only (grade 1), diffuse chorioretinal atrophy (grade 2), patchy chorioretinal atrophy (grade 3), and macular atrophy (grade 4). With increasing MMD severity, the prognosis of visual acuity becomes worse (6, 7). However, the pathogenesis of MMD remains largely unknown.

Oxygen is delivered to the retina from both the retinal and choroidal circulation (8). The balance between retinal and choroidal perfusion plays an important role in several macular diseases (9). Previous studies have shown that the retinal circulation changes with increasing AL, including a reduction in the density of retinal capillary microvasculature and capillary non-perfusion (10, 11). Therefore, in high myopia, MMD caused by chorioretinal atrophy may be associated with a reduced delivery of oxygen caused by the reduced perfusion of the retina.

Cilioretinal arteries, which have a reported prevalence of 20–40% in the normal population, originate from the short posterior ciliary arteries or directly from the choroidal circulation, and usually extend to the macula (12–14). The presence of a cilioretinal artery may afford protection against late age-related macular degeneration (AMD), preventing the development of choroidal neovascularization (15). Therefore, we speculated that cilioretinal arteries, which effectively replace the contribution from central retinal artery (CRA) to the macula area, may also compensate for the reduced blood supply in highly myopic eyes and play a protective role against the development of MMD. However, few relevant studies have been reported.

In the present study, based on fundus photographs taken with an Optos-200Tx device (Optos, Dunfermline, UK), we describe a classification system for cilioretinal arteries that is clinically relevant to the visual function of highly myopic eyes, and demonstrate the protective role of cilioretinal arteries in MMD.

## Materials and Methods

The protocols for this retrospective observational study were approved by the Institutional Review Board of the Eye and Ear, Nose, Throat (EENT) Hospital of Fudan University, Shanghai, China. The study adhered to the tenets of the Declaration of Helsinki. It was affiliated with the Shanghai High Myopia Study, launched at the EENT Hospital of Fudan University in October 2015 (registered at www.clinicaltrials.gov under accession number NCT03062085). Written informed consent for the use of clinical data was obtained from each patient before participation in the study.

### Patients

The Shanghai High Myopia Study is a hospital-based prospective cohort study that continuously includes highly myopic patients aged 18 years or older scheduled for cataract surgery in the EENT Hospital of Fudan University since October 2015. All subjects underwent detailed preoperative examinations and post-operative follow-ups. The medical records of patients included in the Shanghai High Myopia Study database were reviewed. Highly myopic eyes (AL ≥26.0 mm) with clear fundus photographs were included in the study. Eyes with corneal opacity, glaucoma, uveitis, optic nerve disease, fundus pathology other than high-myopia-related changes, previous trauma or vitrectomy, or diabetes were excluded from the study. When both eyes of a patient met the criteria, we randomly selected one eye from each patient. For patients in whom only one eye met the criteria, that eye was included. Ultimately, a total of 1,692 highly myopic eyes of 1,692 patients were included in the study.

### Routine Ophthalmic Examinations

All the eyes included in the study underwent routine ophthalmic examinations, including a slit-lamp examination, funduscopy, fundus photography, B-scan ultrasonography, AL measurement (IOLMaster 700, Carl Zeiss AG, Oberkochen, Germany), and corneal topography. The best-corrected visual acuity [BCVA, logarithm of the minimum angle of resolution (logMAR)] was recorded for each eye included in the study.

### Determination of the Presence of Cilioretinal Arteries and Their Classification

Fundus photographs were collected with the Optos-200Tx ultra-widefield retinal imaging device. Two experienced doctors (WH and JM) independently read all the images to determine the presence of a cilioretinal artery and its classification, before accessing any clinical information. If there was any discrepancy between the two doctors, a senior eye specialist (XZ) gave the final adjudication. A cilioretinal artery was defined as a visible retinal vessel emerging from the border of the optic disc and making a hook-like bend before reaching the retina, with no clear communication with any branches of the central retinal artery. After confirmation of the presence of cilioretinal arteries, their courses, the number of arteries, and distributions were carefully classified.

**Figure 1:**
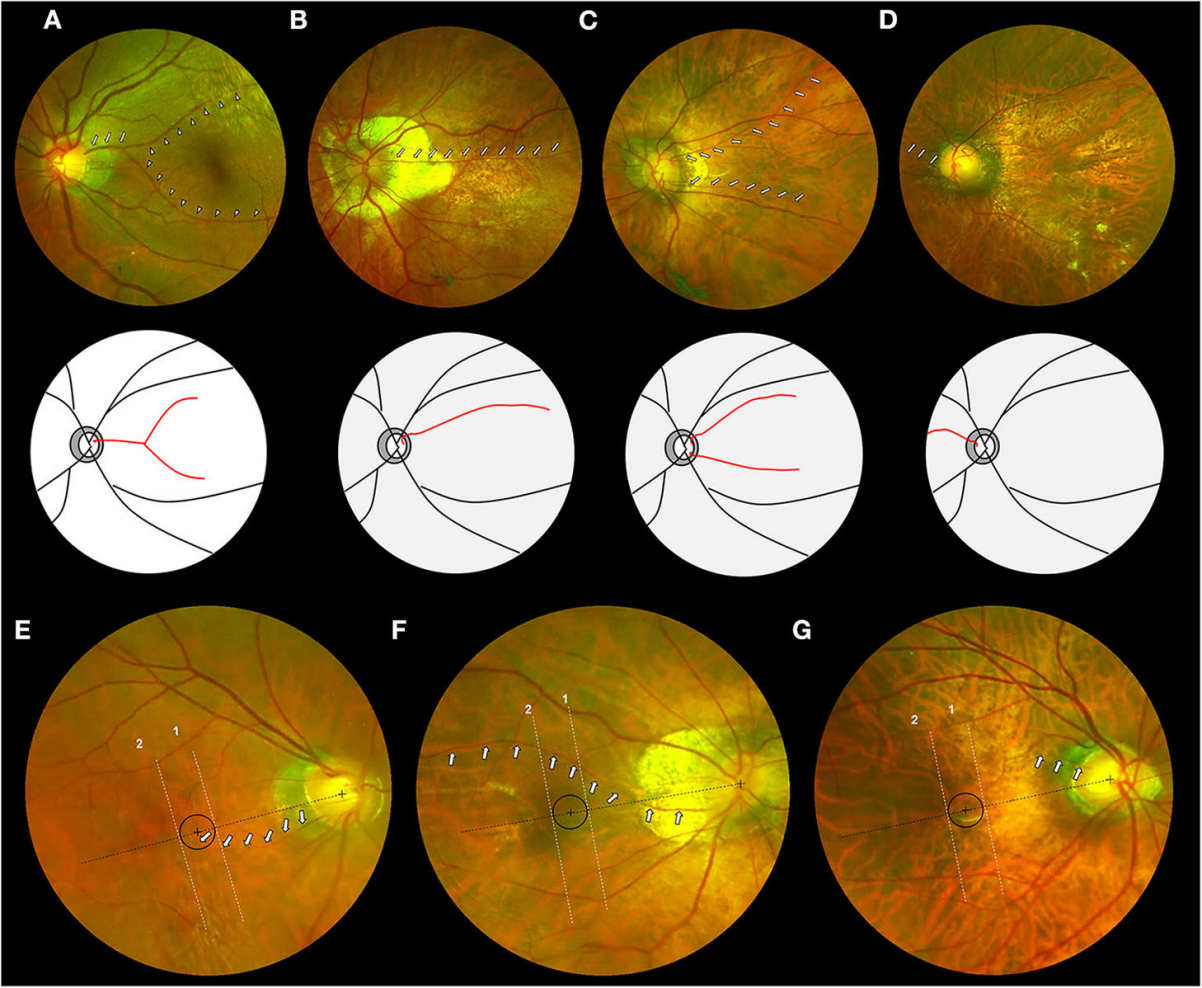
Photographic classification system for cilioretinal arteries in highly myopic eyes. (Top row) Fundus photographs of four categories of cilioretinal arteries. **(A)** Category 1: eyes with a temporal “cake-fork” cilioretinal artery *(white arrows)* with two main branches (*white arrowheads*). **(B)** Category 2: eyes with a temporal “ribbon” cilioretinal artery *(white arrows)*. **(C)** Category 3: eyes with multiple cilioretinal arteries *(white arrows)*. **(D)** Category 4: eyes with a nasal cilioretinal artery *(white arrows)*. (Middle row) Schematic diagrams of four categories of cilioretinal arteries (*red lines*) corresponding to the top row. (Bottom row) Fundus photographs of three distributions of cilioretinal arteries. Black circle represents the area within 500 μm of the foveal center. Lines 1 and 2, both vertical to the dashed black line that connects the centers of the fovea and the optic disc, are used to differentiate the types of distribution. **(E)** Type A: eyes with a cilioretinal artery *(white arrows)* whose path (or visible branches) reaches the area within 500 μm of the foveal center. **(F)** Type B: eyes with a cilioretinal artery *(white arrows)* whose path (or visible branches) extends across the central region. **(G)** Type C: eyes with a cilioretinal artery *(white arrows)* whose path (or visible branches) does not reach the central region.

The cilioretinal arteries were classified into four categories based on the course and number of vessels: Category 1, temporal “cake-fork,” refers to a vessel arising from the temporal disc border and extending like a “cake-fork” with two main branches (Figure 1A); Category 2, temporal “ribbon,” refers to a vessel arising from the temporal border of the disc and extending like a “ribbon” with no obvious branches (Figure 1B); Category 3, “multiple,” refers to two or more cilioretinal arteries per eye (Figure 1C); Category 4, “nasal,” refers to a cilioretinal artery that emerges from the nasal border of the optic disc (Figure 1D). Based on whether it reached the central area within 500 μm of the foveal center (the central circle of the macula grid used in the International META-PM Classification System) (5), the cilioretinal artery was assigned to one of three distributions: Type A (vessel reaching the central area, Figure 1E), Type B (vessel extending across the central area, Figure 1F), or Type C (vessel not reaching the central area, Figure 1G).

### Grading of MMD

The fundus photographs were reviewed and graded by two doctors (WH and JM) according to the International META-PM Classification System (5), without access to any clinical information. Discrepancies were adjudicated by the senior eye specialist (XZ). MMD of grade ≥3 was regarded as severe MMD.

### Statistical Analyses

The results are presented as means ± standard deviations for continuous data and as proportions (%) for categorical data. Interobserver agreement was assessed with the kappa (κ) statistic, and Landis and Koch's (16) guidelines for the interpretation of the κ statistics were applied. Student's *t*-test was used to assess between-group differences in continuous data and a χ^2^-test or Fisher's exact probability test was used to compare categorical data. Analysis of covariance was used to assess the between-group differences in BCVA, with adjustment for AL. Multivariate logistic regression was used to evaluate the contribution of cilioretinal arteries to the MMD grade, with adjustment for AL. A stepwise multivariate linear regression analysis was used to identify the effects of age, sex, eye laterality, AL, and the presence of a cilioretinal artery on BCVA. By using the stepwise regression mode, eye laterality was removed from the model while the other independent variables were included in the regression model. For eyes with cilioretinal arteries, one-way ANOVA with Tukey's *post-hoc* test was used to compare continuous data among different categories or distributions. All statistical analyses were performed with SPSS version 22 (SPSS, Chicago, IL, USA). Two-sided *P* < 0.05 were considered statistically significant in all analyses.

## Results

### Characteristics

**Figure 2:**
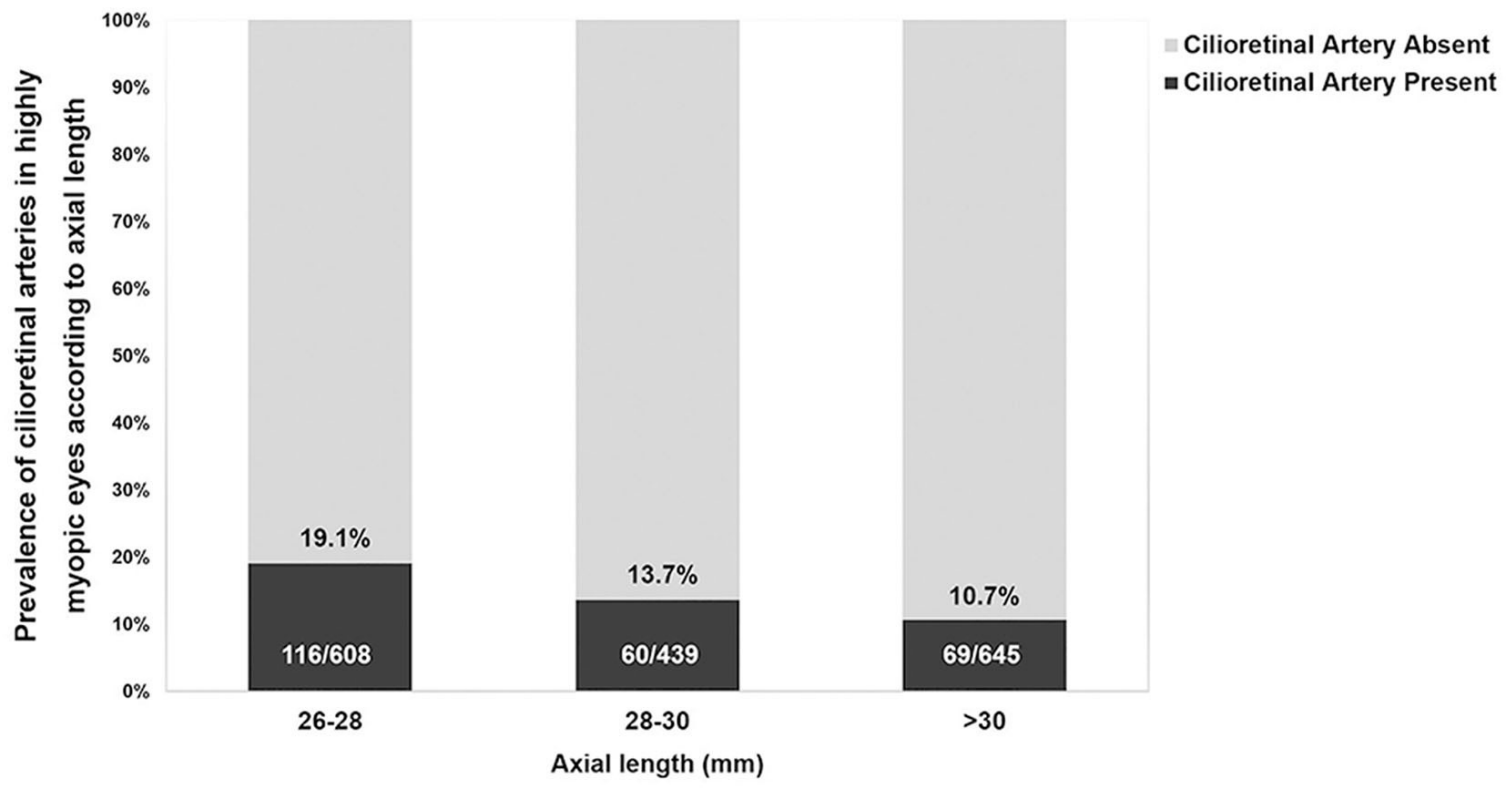
Prevalence of cilioretinal arteries in highly myopic eyes with different axial lengths.

The mean age of the 1,692 patients analyzed was 61.19 ± 9.12 years, and 42.7% (723/1,692) were male. The mean AL was 29.35 ± 2.30 mm, with a range of 26.01–36.50 mm. Of all the eyes included, 14.5% (245/1,692) had a cilioretinal artery (interobserver agreement was 98.05% and κ was 0.92 [95% confidence interval: 0.90–0.95]). Table 1 lists the characteristics of the highly myopic eyes with and without cilioretinal arteries. No significant differences in age, sex, or eye laterality were detected between the two groups (Student's *t*-test for age and χ^2^-test for sex and eye laterality; all *P* > 0.05). Eyes with cilioretinal arteries were shorter than those without (cilioretinal artery present vs. absent: 28.86 ± 2.16 vs. 29.43 ± 2.31 mm, respectively; Student's *t*-test, *P* < 0.001). With increasing AL, the prevalence of cilioretinal arteries in the highly myopic eyes decreased, from 19.1% in the 26–28 mm AL group to 10.7% in the >30 mm AL group (Figure 2).

**Table 1:** Baseline characteristics.

	**Cilioretinal artery present (*n* = 245)**	**Cilioretinal artery absent (*n* = 1,447)**	***P*-value**
Age^*^, years	61.71 ± 9.03	61.10 ± 9.13	0.328
Sex^†^, male/female	116/129	607/840	0.114
Eye laterality^†^, right/left	113/132	738/709	0.158
AL^*^, mm	28.86 ± 2.16	29.43 ± 2.31	<0.001

*AL, axial length*.

* *Student's t-test*.

† *χ^2^-test*.

### Photographic Classification System for Cilioretinal Arteries in Highly Myopic Eyes

As to the photographic classification, the percentages of the temporal “cake fork,” the temporal “ribbon,” the “multiple,” and the “nasal” categories were 35.92% (88/245), 53.47% (131/245), 6.53% (16/245), and 4.08% (10/245), respectively. The cilioretinal artery had three main types of distribution, based on whether its path or visible branches reached the area within 500 μm of the central fovea. The percentage of Type A (arteries whose paths or visible branches reached the area within 500 μm of the foveal center), Type B (long vessels whose paths or visible branches extended across the central region), and Type C (vessels whose paths or visible branches did not reach the central region) were 37.96% (93/245), 24.49% (60/245), and 37.55%(92/245), respectively. Excellent agreement between the observers was observed, with κ ≥ 0.87 (see Supplementary Table 1).

### Association Between the Presence of a Cilioretinal Artery and MMD or Visual Acuity

Of all the eyes, 76.5% (1,295/1,692) were classified with grade 1 or grade 2 MMD, 17.7% (299/1,692) had grade 3, and 5.8% (98/1,692) had grade 4. The interobserver agreement in the MMD grading was excellent, with κ-values of 0.88–0.96 (see Supplementary Table 1). Table 2 lists the MMD grades for the highly myopic eyes with and without cilioretinal arteries. Because of the possible correlations between AL and MMD grade, we used a multivariate logistic regression analysis adjusted for AL and found that significantly more eyes with grade 1 MMD were found in eyes with cilioretinal arteries than in those without (*P* < 0.001). Both rates of grade 3 and grade 4 MMD were significantly lower in eyes with cilioretinal arteries than in those without after adjustment for AL (*P* = 0.003 and *P* = 0.006, respectively).

**Table 2:** MMD grading of highly myopic eyes with and without cilioretinal arteries.

**Grade**	**Cilioretinal artery present, *N* (%)**	**Cilioretinal artery absent, *N* (%)**	*P*-value^*^
Grade 1	148 (60.41%)	582 (40.22%)	<0.001
Grade 2	73 (29.79%)	492 (34.00%)	0.494
Grade 3	22 (8.98%)	277 (19.14%)	0.003
Grade 4	2 (0.82%)	96 (6.64%)	0.006

*MMD, myopic macular degeneration; N, number*.

* *Multivariate logistic regression with adjustment for axial length*.

We used a covariance analysis for BCVA because of the possible correlation between AL and visual acuity, and found that eyes with cilioretinal arteries showed better BCVA than those without, after adjustment for AL (cilioretinal artery present vs. absent: 0.16 ± 0.21 logMAR [Snellen 20/29] vs. 0.24 ± 0.33 logMAR [Snellen 20/35], respectively; *P* = 0.007). A backwards stepwise multiple linear regression analysis showed that younger age (β = 0.004, *P* < 0.001), male sex (β = 0.045, *P* = 0.002), shorter AL (β = 0.044, *P* < 0.001), and the presence of a cilioretinal artery (β = −0.055, *P* = 0.007) were all associated with better BCVA in highly myopic eyes.

### Associations Between the Classification of Cilioretinal Arteries and MMD or Visual Acuity

**Figure 3:**
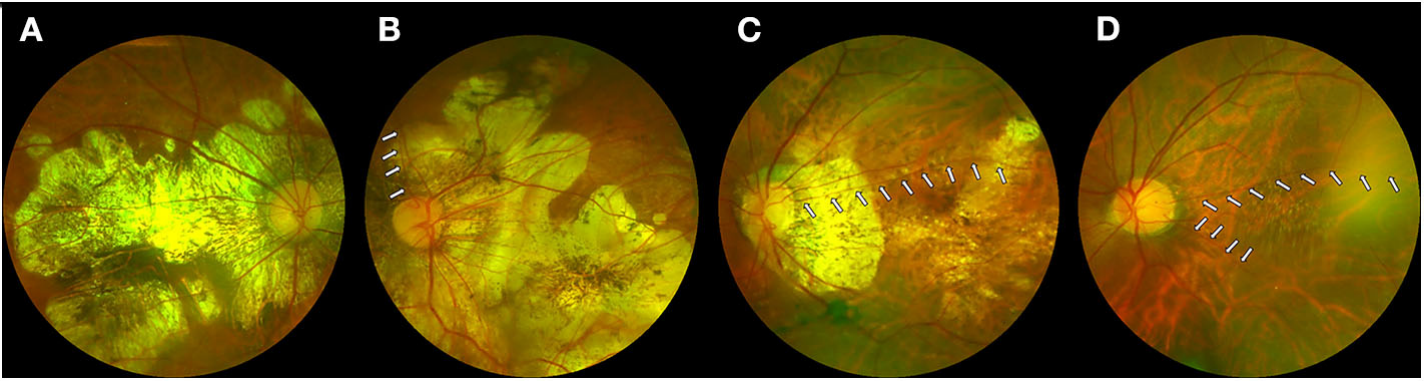
Myopic macular degeneration in eyes with or without cilioretinal arteries. **(A)** Eyes without a cilioretinal artery. **(B)** Eyes with a nasal cilioretinal artery (*white arrows*). **(C)** Eyes with a temporal cilioretinal artery (*white arrows*). **(D)** Eyes with multiple cilioretinal arteries (*white arrows*).

Table 3 lists the MMD grades and BCVAs for the highly myopic eyes in different categories and with different distributions of cilioretinal arteries. AL did not differ among the four categories or among the three distributions. Among the eyes with cilioretinal arteries, the “nasal” category (Category 4) presented more MMD with grade ≥3 than the other categories (χ^2^-test, *p* = 0.002; Table 3). No difference was identified in severe MMD between eyes with nasal cilioretinal arteries and those without a cilioretinal artery (χ^2^-test, *p* = 0.138). No eyes with a Category 3 cilioretinal artery had MMD grade ≥3. In terms of visual acuity, the “nasal” category (Category 4) had worse BCVA than those in the other categories (one-way ANOVA with Tukey's *post-hoc* test, *P* < 0.001; Table 3). Figure 3 shows that eyes without a cilioretinal artery or with a nasal cilioretinal artery were more likely to display macular atrophy than those with temporal or multiple cilioretinal arteries. In terms of the distribution of cilioretinal arteries, significantly fewer eyes with severe MMD (grade ≥3) were identified as Type A than as Type C. The percentage of eyes with MMD grade ≥3 was 4.30% for Type A and 15.22% for Type C (χ^2^-test, *P* = 0.012). Type A also had significantly better BCVA than Type C (0.12 ± 0.17 logMAR [Snellen 20/26] vs. 0.20 ± 0.25 logMAR [Snellen 20/32], respectively; one-way ANOVA with Tukey's *post-hoc* test, *P* = 0.026).

**Table 3:** MMD grading and BCVAs in eyes with different categories and distributions of cilioretinal artery.

	MMD grading^*^			**BCVA^†^, logMAR**
**Classification**	**Grades ≤2, *N* (%)**	**Grade 3, *N* (%)**	**Grade 4, *N* (%)**	
**Category**				
Category 1	83 (94.32%)	5 (5.68%)	0 (0%)	0.12 ± 0.14
Category 2	117 (89.31%)	14 (10.69%)	0 (0%)	0.16 ± 0.20
Category 3	16 (100%)	0 (0%)	0 (0%)	0.12 ± 0.17
Category 4	5 (50.00%)	3 (30.00%)	2 (20.00%)	0.59 ± 0.43
P value	0.002	0.051	–	<0.001
**Distribution**				
Type A	89 (95.70%)	4 (4.30%)	0 (0%)	0.12 ± 0.17
Type B	54 (90.00%)	6 (10.00%)	0 (0%)	0.16 ± 0.20
Type C	78 (84.78%)	12 (13.04%)	2 (2.18%)	0.20 ± 0.25
*P*-value	0.044	0.109	–	0.035
Cilioretinal artery absent	1,074 (74.22%)	277 (19.14%)	96 (6.64%)	0.24 ± 0.43

*MMD, myopic macular degeneration; BCVA, best-corrected visual acuities; logMAR, logarithm of the minimum angle of resolution; N, number*.

* *χ^2^-test or Fisher's exact probability test*.

† *One-way ANOVA with Tukey's post-hoc test*.

## Discussion

In recent years, MMD has become one of the major threats to visual health worldwide (17–19). MMD is aggravated by the reduced retinal microvasculature that occurs with elongation of the eyeball (20–22), suggesting a role for reduced retinal perfusion in the development of MMD. The cilioretinal artery may contribute to the blood supply to the macula and can prevent the progression of fundus diseases such as AMD (15). However, there have been few observations on the role of cilioretinal artery in MMD of highly myopic eyes and its classifications as well. Therefore, in the present study we first presented a classification system for cilioretinal arteries in highly myopic eyes based on a large sample of fundus photographs, and then analyzed its associations with MMD and visual acuity and found that the cilioretinal artery is a protective factor against MMD.

The cilioretinal artery, initially described by Mueller (23), is a retinal artery that may have a protective effect in fundus diseases (15, 24–26). In central retinal artery occlusion, eyes with cilioretinal arteries can show macular sparing and gain an intact central island field (24). The presence of a cilioretinal artery is also recognized as a protective factor in AMD (15). However, little is known about the effect of this vessel on MMD. To our knowledge, this study is the largest investigation until now of cilioretinal arteries in highly myopic eyes, and reports a prevalence of 14.5%. The prevalence of cilioretinal arteries decreases as AL increases, suggesting that the contribution from cilioretinal arteries to the macula tends to be weaker in longer eyes.

Because the anatomical features of the fundus are complex in highly myopic eyes (5, 27), the courses of cilioretinal arteries vary greatly. Therefore, a standard classification system is required to facilitate clinical and epidemiological studies of cilioretinal arteries. Based on the course, number, and distribution of cilioretinal arteries in highly myopic eyes, we propose a photographic classification system that includes four categories and three distributions. In highly myopic eyes, the most frequent category was the temporal “ribbon,” followed by the temporal “cake-fork.” The percentages of eyes in the “multiple” and “nasal” categories in our study were 6.53 and 4.08%, respectively, which are similar to the 7 and 6%, respectively, in the normal population (28).

In this study, a protective role for the cilioretinal artery in high myopia was identified for the first time. After adjustment for AL, the eyes with cilioretinal arteries had a lower prevalence of severe MMD, especially vision-threatening macular atrophy. Moreover, the severity of MMD and the function of the macula are directly related to the visual acuity of patients with high myopia (6, 7). Generally, eyes with cilioretinal arteries showed better visual acuity than those without, and our multivariate regression analysis confirmed that the presence of a cilioretinal artery had a significantly favorable effect on visual acuity.

On the one hand, the cilioretinal artery may protect the macula by compensate for the hemodynamic imbalance of the retina. In high myopia, the main clinical features occur at the level of RPE and choroid (5), which may influence the perfusion from the choroid to the macula and disrupt the hemodynamic balance between the retinal and choroidal circulation. The cilioretinal artery may partly compensate for the hemodynamic imbalance and prevent the development of macular degeneration (15). On the other hand, retina vessels (irrespective of their origin from the CRA or cilioretinal artery) are basically end arteries with no anastomosis between them. Besides, the CRA and cilioretinal vessels supply distinct areas within the macula, which is supported by the clinical observation that the cilioretinal artery occlusion usually shows the same degree of retinal disability as the occlusion of a branch of CRA (24). Thus, the cilioretinal arteries do not increase the blood supply but effectively replace the contribution from the CRA to a variable segment of the retina. We understand that the ciliary circulation is influenced by sympathetic innervation while the retinal circulation has the autoregulatory mechanism (29). Also, different clinical features between eyes with cilioretinal arteries and those without have been reported in some fundus diseases, such as choroidal neovascularization, giant cell arteritis, and retinal angiomatous proliferation (15, 30, 31). Yet, it remains unclear whether in highly myopic eyes, the area of retina perfused by cilioretinal arteries behaves different from that perfused by the CRA. Therefore, future observation is needed to definitely map the area supplied by the cilioretinal artery and determine its correlation with the area of MMD based on OCT angiography.

Our photographic classification system also showed good clinical relevance to the patients' visual function. We found that nasal cilioretinal arteries presented more severe MMD than the other categories of cilioretinal arteries, but no difference was identified in severe MMD between eyes with nasal cilioretinal arteries and those without a cilioretinal artery. This indicated that the “nasal” category may have little influence on the development of MMD. On the contrary, in eyes in the “multiple” category, more protection seemed to be provided to the macula insofar as no eye in this category was identified with severe MMD. The temporal “cake-fork” which includes superior and inferior branches and provides perfusion to a wider area than the temporal “ribbon,” may take second place. In terms of the distributions of the arteries, whether the end of the cilioretinal artery reaches the key area within 500 μm of the central fovea may be highly clinically significant. In Type A, the rate of severe MMD was low (4.30%), and no macular atrophy was identified. In contrast, the protective effect of the artery decreased if the vessel was either too short or too long. Although the long Type B vessels extended across the macula, the actual area of effective perfusion may have been located at the far end of the vessel.

In this study, fundus photography was used to determine the presence of cilioretinal arteries and their classifications. Although fluorescence angiography was recognized as a more precise way to evaluate the cilioretinal arteries, fundus photography may be more convenient for the rapid screening and daily clinical application. Most importantly, it is non-invasive. However, to further validate the results obtained from the fundus photo, future study based on non-invasive imaging modalities like OCT angiography are still needed. In addition, reaching central 500 microns area is not the same as actually participating in the peri FAZ ring of capillaries. Very often that decides the macular sparing effect of cilioretinal artery during CRVO. Therefore, based on OCT angiography analysis, the role of the cilioretinal artery participating in the peri FAZ ring of capillaries should be analyzed. Another limitation of this study is the retrospective design including only one single institution. Further prospective studies are needed to confirm the generalizability of the current results.

In conclusion, based on fundus photographs, we propose a classification system for cilioretinal arteries that has good clinical relevance to visual functions, and have demonstrated that the cilioretinal artery is a potentially protective factor against MMD and loss of visual acuity in highly myopic eyes.

## Data Availability Statement

The raw data supporting the conclusions of this article will be made available by the authors, without undue reservation.

## Ethics Statement

The studies involving human participants were reviewed and approved by the Institutional Review Board of the Eye and Ear, Nose, Throat (EENT) Hospital of Fudan University, Shanghai, China. The patients/participants provided their written informed consent to participate in this study.

## Author Contributions

XZ and YL: conception and design. XZ, JM, and LW: analysis and interpretation of data and manuscript preparation. JM, LW, KZ, and WH: data collection. All authors contributed to the article and approved the submitted version.

## Conflict of Interest

The authors declare that the research was conducted in the absence of any commercial or financial relationships that could be construed as a potential conflict of interest.
