# Supplementary material for: Cilioretinal Arteries in Highly Myopic Eyes: A Photographic Classification System and Its Association With Myopic Macular Degeneration
Source: Front Med (Lausanne). 2020 Dec 2;7:595544. doi: 10.3389/fmed.2020.595544 (PMC7738318; doi:10.3389/fmed.2020.595544)
Supplement: Supplementary file 1 [file Table_1.DOCX]

**Supplementary Table**

Inter-observer agreement on the classification of cilioretinal artery and MMD grading

|  | Inter-observer agreement | |
| --- | --- | --- |
|  | Agreement (%) | Kappa (±SE) |
| Category | | |
| Category 1 | 93.88 | 0.88(0.03) |
| Category 2 | 94.29 | 0.88(0.03) |
| Category 3 | 98.78 | 0.90(0.06) |
| Category 4 | 99.18 | 0.91(0.07) |
| Distribution | | |
| Type A | 95.10 | 0.89(0.03) |
| Type B | 95.51 | 0.88(0.04) |
| Type C | 93.88 | 0.87(0.03) |
| MMD grading | | |
| Grade 1 | 98.11 | 0.96(0.01) |
| Grade 2 | 97.34 | 0.94(0.01) |
| Grade 3 | 98.17 | 0.94(0.01) |
| Grade 4 | 98.64 | 0.88(0.02) |
| MMD = myopic macular degeneration; SE = standard error. | | |
